# Psychometric Properties of Diabetes Management Self-Efficacy in Thai Type 2 Diabetes Mellitus Patients: A Multicenter Study

**DOI:** 10.1155/2017/2503156

**Published:** 2017-10-22

**Authors:** Monthida Sangruangake, Chananya Jirapornkul, Cameron Hurst

**Affiliations:** ^1^Public Health Program, Khon Kaen University, Khon Kaen, Thailand; ^2^Faculty of Nursing, Ratchathani University, Udonthani, Thailand; ^3^Department of Epidemiology and Biostatistics, Faculty of Public Health, Khon Kaen University, Khon Kaen, Thailand; ^4^Center of Excellence in Biostatistics, Faculty of Medicine, Chulalongkorn University, Bangkok, Thailand

## Abstract

**Objective:**

The aims of this study were to translate and psychometrically evaluate the Thai version of diabetes management self-efficacy scale (T-DMSES) and to examine its association with HbA1c control in diabetic individuals.

**Methods:**

This study recruited patients from outpatient diabetes clinics of both community and university hospitals. The first phases of this study involved translation of the existing DMSES into Thai, and in the second phase, we evaluated its psychometric properties. The construct validity was evaluated using confirmatory factor analysis. Criterion validity of DMSES was subsequently evaluated by examining DMSES's association with HbA1c control.

**Results:**

The T-DMSES contains 20 items across four factors. Confirmatory factor analysis demonstrated the construct validity of T-DMSES (*χ*^2^ = 645.142, df = 164, *p* < 0.001, CFI = 0.98, RMSEA = 0.065, TLI = 0.977, and AGFI = 0.981). The T-DMSES was also shown to be criterion valid with most subscales highly associated with HbA1c control.

**Conclusion:**

The T-DMSES was shown to have good psychometric properties. It is likely to provide valuable insights into the epidemiology of diabetes management self-efficacy and may also prove useful in evaluating interventions for raising diabetes management self-efficacy, which in turn, improve both patient self-management and blood sugar control.

## 1. Introduction

Diabetes represents a major burden, and it is estimated that approximately 366 million people currently live with this condition worldwide and that the prevalence of diabetes will increase considerably in the coming decades [1]. Although the prevalence of diabetes in most Asian countries, especially lower and middle income like Thailand, is lower than in western countries, diabetes still represents a major cause of morbidity and mortality; furthermore, the prevalence of diabetes in Thailand is increasing at an alarming rate [2]. Currently, there are 3.2 million patients with diabetes in Thailand, and this is estimated to rise by 1.1 million patients by 2035 [3].

Type 2 diabetes mellitus (T2DM) is a disease where patient self-care can substantially reduce the risk or delay the onset of chronic T2DM complications such as cardiovascular disease, nephropathy, retinopathy, and neuropathy, all of which lead to substantial morbidity and mortality [4]. However, unlike many other chronic diseases, diabetes progression can be slowed considerably by patients' adherence to taking medications as prescribed, monitoring their diet and blood glucose levels, engaging in physical activity, and caring for their feet [5]. Such activities need to be incorporated into daily life allowing patients to better control their disease and prolong the onset of complications [6]. The above process is defined as diabetes self-management (DSM). Successful diabetes management relies on the long-term cooperation of patients in obtaining regular medical care and adhering to treatment plans [7–9].

Diabetes education has traditionally emphasized positive change through improving patient's knowledge and attitude. However, it is insufficient to realize desirable self-care behaviors in diabetes patients from knowledge and attitude changes through information transfer and instruction alone [10]. Self-efficacy is a factor considered to be of particular importance to the process of changing self-care behavior and personal health outcomes [11].

Self-efficacy refers to an individual's perceptions or beliefs in their capabilities to carry out certain activities. It is influential to their thinking, feeling, motivation, and behaviors. Those with high self-efficacy choose to perform more challenging tasks. They set higher goals for themselves and adhere to these goals more effectively [12]. To take an action, individuals who have high self-efficacy exert more effort with more persistence than those who have low self-efficacy. In case of setbacks, they achieve quicker recovery and continue the commitment to achieving their goals. This efficacious outlook contributes to personal accomplishments and stress reduction [13]. Several studies have presented the effect of self-efficacy on self-care behaviors and clinical outcomes in patients with diabetes. Previous research demonstrates that self-efficacy shows a significant relationship with management of drug therapy, diet, physical activity, blood glucose monitoring, and HbA1c control in T2DM patients [14–18].

The diabetes management self-efficacy scale (DMSES) was originally developed for use in western populations and assesses the confidence of diabetes patients in their ability to manage their diet, blood sugar, and physical exercise [19]. To date, the instrument has been widely recognized, adapted, and translated for use in several countries representing a wide range of healthcare settings including the United Kingdom [20], Australia [8], Turkey [21], Greek [22], Iran [23], Taiwan [24], and Korea [25]. However, a comparison of these studies suggests differences in the relative importance of the individual components of diabetes management self-efficacy and these differences may result from disparity in the study population, healthcare setting, or sampling design. Consequently, the application of the original version of the DMSES instrument has varied across studies as evidenced by the different numbers and natures of factors generated [21, 23, 24].

There are also some methodological limitations in many of the previous validation studies of DMSES. The methodological approaches used in most previous studies have typically been either incomplete or, in some cases, even inappropriate. Many studies have employed exploratory factor analysis using principal component analysis with orthogonal rotation suggesting a belief that the underlying constructs of DMSES are uncorrelated, which is something unlikely to be true. Also, exploratory factor analysis alone provides insufficient evidence of construct validity, and principal component analysis is widely recognized as a poor method for elucidating factorial structure [26]. Indeed, to date, DMSES has only been structurally validated in one population elsewhere, the Korean T2DM population [25]. In the present study, we assess both the construct and criterion validity of the DMSES in Thai T2DM patients and investigate how DMSES is associated with patients' characteristics.

## 2. Method

This two-phase study was designed to evaluate the psychometric properties of Thai diabetes management self-efficacy scale (T-DMSES). Phase I involved the translation of the existing a Dutch/English version of DMSES [19] into Thai, thereby establishing translational validity. Phase II was concerned with evaluating the psychometric properties of the T-DMSES.

### 2.1. Phase 1: Instrument Translation and Face Validity

The original version of the DMSES is a self-administered scale composed of 20 items designed to investigate T2DM management self-efficacy in terms of four factors, namely, specific nutrition and weight, general nutrition and medical treatment, physical exercise, and blood sugar monitoring [19]. In our study, the DMSES items were translated from English into Thai using the forward and backward translation technique outlined by Brislin [27]. Four Thai-English bilingual translators were identified, and of these, two were used to forward translate the original version of the DMSES instrument into Thai, while the remaining two translators were used to back-translate the instrument from Thai to English. The original and back-translated versions of DMSES were then compared by two native English speakers. Finally, the T-DMSES was field-tested in a pilot group consisting of 20 Thai T2DM patients to evaluate the translational quality and the practical aspects of test administration. Participants were asked to read and listen to each item in order to ensure their understanding.

### 2.2. Phase 2: Evaluating the Psychometric Properties of the T-DMSES

#### 2.2.1. Sample and Study Design

This cross-sectional study was used to evaluate the psychometric properties of DMSES in Thai T2DM patients. In total, 700 T2DM patients living in either rural or urban areas from the central and northeastern regions of Thailand were recruited from outpatient diabetes clinics of both community and university hospitals in both the Khon Kaen and Bangkok provinces of Thailand. Patients were sampled using a stratified sampling design where strata were based on locality (province)—hospital size combinations. The questionnaire was administered in February to June 2016 to T2DM outpatients aged ≥ 20 years old who had had a diagnosis of T2DM for at least 3 years, were able to read and understand the Thai language, and were willing to participate in the study. The authorized person of each hospital gave permission to collect the data, and all participants provided written informed consent. Participants were collected until the data collection was completed by means of a participant self-report. The study protocol was approved by the ethics committee of Khon Kaen University (HE581479), Institutional Review Board at Faculty of Medicine, Chulalongkorn University (IRB035/59), and the Bangkok Metropolitan Administration Ethics Committee for Human Research (U005q/59).

#### 2.2.2. Measurement

The T-DMSES is an instrument that aims to measure the diabetes management self-efficacy in Thai T2DM patients. This instrument's 20 items are distributed across 4 factors as follows: *diet* (9 items), *monitor* (4 items), *physical* (4 items), and *regimen* (3 items). All items are rated on a 5-point Likert scale from strongly disagree to strongly agree. Our questionnaire also included 14 questions relating to sociodemographics including gender, marital status, age, education, religion, household income, weight, height, family history of T2DM, smoking, and alcohol consumption. In addition, comorbidities, duration of diabetes, type of diabetes treatment, and glycated hemoglobin (HbA1c), an indicator of glycemic control, were extracted from each patient's electronic medical records using the most recent visit for each participant.

#### 2.2.3. Statistical Analysis

Patient characteristics were summarized using descriptive statistics, with means and standard deviations used for continuous variables and counts and percentages for categorical data.

**Figure 1:**
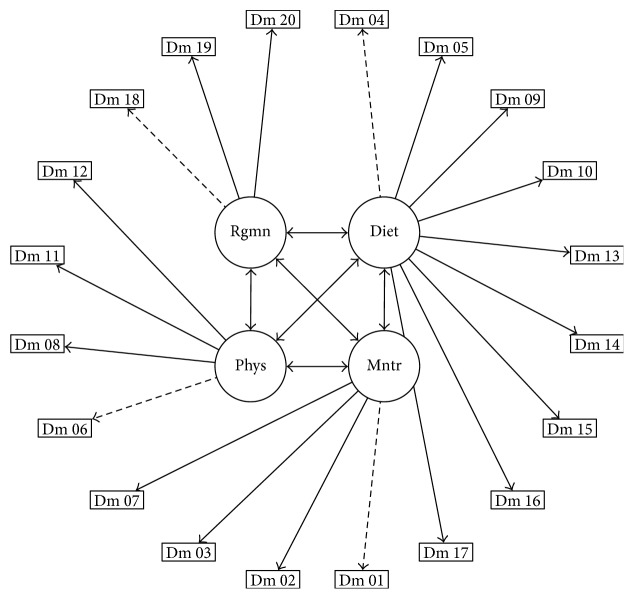
The measurement model for the CFA of T-DMSES based on a four-factor structure including regimen self-efficacy (regimen), diet management self-efficacy (diet), physical activity self-efficacy (physical), and monitoring self-efficacy (monitor).

For the DMSES measurement model, we specified the model as identified by several previous studies [8, 20–25]. In particular, we followed the structure identified in the Chinese population of Taiwan [24] based on the comparative similarity of the Chinese and Thai populations. The measurement model is provided in Figure 1. An unweighted least square confirmatory factor analysis (CFA) was used to fit the measurement model, and model fit was assessed using the cumulative fit index (CFI), adjusted goodness of fit index (AGFI), root-mean-square error of approximation (RMSEA), and the Tucker-Lewis index (TLI). A model with TLI, CFI [28], GFI [29], and AGFI > 0.9 [30] and RMSEA < 0.08 [31] was deemed to represent adequate model fit. We also reported the *χ*^2^ statistics, typically a poor indicator of measurement model fit but included here for reasons of convention. Bartlett's test of sphericity and the Kaiser-Meyer-Olkin (KMO) measure of sampling adequacy were generated along with the CFA to provide further evidence of construct validity [32].

Criterion validity was assessed based on the T-DMSES's ability to discriminate between patients with good and poor HbA1c control. Receiver operating characteristic (ROC) curves along with the sensitivity, specificity, positive and negative predictive values, and positive and negative likelihood ratios were used to gauge criterion validity. Internal consistency reliability was evaluated using Cronbach's alpha, and an acceptable reliability was considered to be *α* > 0.7 for all the subscales [33]. Finally, how the T-DMSES subscales might be associated with patient characteristics was investigated using ordinal logistic regression. For this analysis, each subscale (diet, monitor, physical, and regimen) was collapsed into a three-point ordinal scale with the lowest group represented by those at least one standard deviation below the mean (approximately 16%); the middle group, those within one standard deviation of the mean (approximately 68%); and the highest, those at least one standard deviation above the mean (approximately 16%). The R statistic package (version 3.2.0; R CoreTeam, 2015) was used to conduct all analysis, and the R library lavaan was used for factor analysis [34]. The ROC curves and corresponding statistics were generated using the R library Epi [35]. A significance level of 0.05 was used throughout all inferential analysis.

## 3. Result

### 3.1. Demographic Characteristics of Participants

In total, 700 T2DM patients completed the questionnaire (response rate of 100%), with ages ranging from 26 to 95 years old (mean = 65.16, SD = 10.94). The sample consisted of 70.29% females, and the average number of years since T2DM diagnosis was 13.53 years (SD = 8.34). The demographic characteristics of the participants are presented in Table 1.

### 3.2. Psychometric Properties of T-DMSES

The T-DMSES measurement model was represented by 20 items distributed across four factors and was fit using an unweighted least square confirmatory factor analysis. The measurement model for the CFA is shown in Figure 1. Based on the five preestablished fit criteria, the model showed adequate fit to the data (*χ*^2^ = 645.142, df = 164, and *p* < 0.001; CFI = 0.98; RMSEA = 0.065; TLI = 0.977; and AGFI = 0.981). All items in the model loaded significantly on their respective factors (all *p* < 0.05) except each factor-constraint item for which no significance test could be conducted. The resulting standardized factor loadings are presented in Table 2, and the interfactor correlations are given in Table 3. The KMO was 0.88, and Bartlett's sphericity test was significant (*χ*^2^ = 8576.884, df = 190, and *p* < 0.001) indicating reasonable adequacy of the data for factor analysis.

The interfactor correlations of the T-DMSES subscales are presented in Table 3 and illustrate that diet was strongly positively associated with monitor and moderately positively associated with physical. Monitor was also moderately positively associated with physical (Table 3).

**Figure 2:**
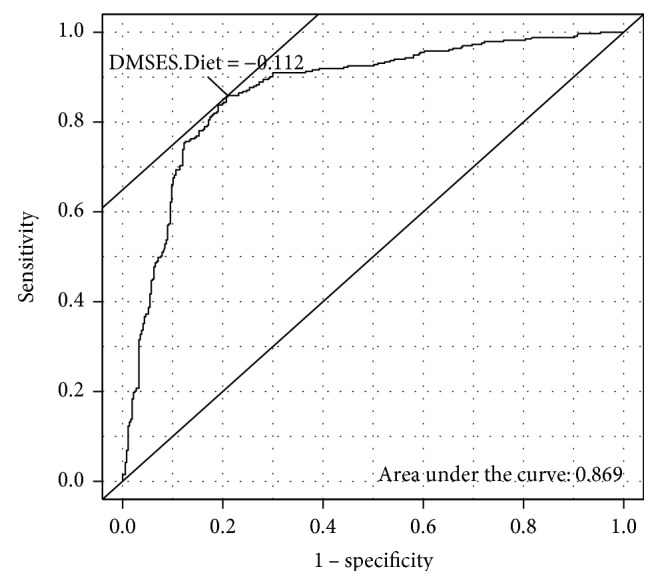
Receiver operating characteristic curve for the diet subscale of T-DMSES against HbA1c control.

Criterion validity was assessed based on the T-DMSES subscale association with a concurrent measure of the HbA1c clinical target (controlled: HbA1c ≤ 7%; uncontrolled: HabA1c > 7%). The sensitivity, specificity, positive and negative predictive value, and positive and negative likelihood ratios of each subscale and the overall T-DMSES scale are presented in Table 4. Diet in particular is shown to be quite effective in discriminating between those with and without concurrent blood sugar control. The ROC curve of the diet subscale is provided in Figure 2.

Cronbach's alpha demonstrated that the T-DMSES achieved a good level of internal consistency reliability with Cronbach's alpha of 0.89 for the overall scale. The Cronbach alpha values for the subscales are presented in Table 5. Perusal of Table 5 suggests that all subscales had good internal consistency reliability with the exception of monitor whose Cronbach's alpha was 0.45.

We also investigated whether the various T-DMSES subscales are associated with patient characteristics. The results of this analysis are presented in Table 6. Those who are living in Khon Kaen or had a history of alcohol consumption have a higher probability of poorer diet and regimen self-efficacy, whereas female and older patients demonstrated better diet and regimen self-efficacy. Also, family history of diabetes and a higher level of education (relative to no formal education) were significantly related to poorer diet self-efficacy. Small (community) hospital and higher income are associated with poorer regimen self-efficacy, whereas longer duration of T2DM was associated with better regimen self-efficacy. Patients with BMI < 18.5 kg/m^2^ or those who were current smokers tended to achieve better monitor self-efficacy. In contrast, those receiving prescribed treatments (e.g., oral hyperglycemic agents, insulin, or oral hyperglycemic agents, and insulin) had poorer monitor self-efficacy.

## 4. Discussion

T2DM is a chronic illness associated with numerous comorbidities, and this disease leads to chronic complications, resulting in high morbidity and mortality and raising healthcare costs. However, patients with this disease, through self-care, can significantly mitigate the risk or delay the onset of T2DM complications. In this study, we translate and validate the DMSES in the Thai T2DM patient population. The diabetes management self-efficacy scale (DMSES) [19] is one of the most widely used instruments to measure diabetes management self-efficacy. However, even though several studies have adapted and translated the DMSES to be used across several healthcare setting and populations [8, 20–25], its use in these settings has generally not been supported by a prior appropriate validation. Indeed, to the best of our knowledge, only one previous study has conducted a confirmatory factor analysis on the DMSES, that of Lee et al. [25], and in this respect, the construct validity of DMSES has not been adequately established in a large majority of the studies that have employed it.

In the construct validation in the present study, we tested the structure of the DMSES identified in the Chinese population in Taiwan [24] and established that the measurement model of the Thai version of the DMSES fits the data well. With regard to criterion validity, we demonstrated that all T-DMSES subscales are strongly associated with the clinical outcome, HbA1c control. This result is supported by Sturt and colleagues who also identified a strong association between HbA1c and the overall DMSES score [20]. In the present study, we found that the DMSES subscale, diet self-efficacy in particular, is quite effective at discriminating between those with good and poor blood sugar control. Norris and colleagues reported that diet control is an essential part of therapy for a patient with T2DM [11]. Effective nutrition intervention in T2DM care management has been shown to contribute to improve HbA1c control, resulting in decreased medication, frequency of hospitalization, and overall healthcare costs [36, 37]. Interventions that can improve diabetes management self-efficacy may have a major impact on the patients' ability to achieve one of the most important type 2 diabetes clinical targets, HbA1c control, a clinical target whose control is well established as protective against the development of T2DM chronic complications.

In terms of the internal consistency reliability of the T-DMSES and its subscale, we demonstrated strong internal consistency for the T-DMSES and all but one of its subscales. The monitor DMSES subscale had comparatively poor internal consistency (Cronbach's alpha = 0.45) suggesting that at least some of the monitor items were not highly correlated with each other. Interestingly, this low degree of internal consistency does not seem to interfere with the monitor subscale's ability to discriminate patients who do and do not control their blood sugar. The sensitivity and specificity of the monitor subscale for predicting HbA1c control were 0.83 and 0.76, respectively.

We also investigated patient characteristics that may be associated with diabetes self-efficacy. Provinces, hospital size, gender, age, level of education, income, BMI, family history of diabetes, type of treatment, alcohol drinking, duration of T2DM, and smoking were all found to be associated with various T-DMSES subscales. These results are supported by several studies [38–42]. Interestingly, the present study could not demonstrate that particular patient characteristics were consistently associated across all, or even most, DMSES subscales, suggesting that the associations of different patient characteristics with different aspects of diabetes management self-efficacy are relatively complex; different subgroups of patients are likely to be at risk of different aspects of poor diabetes management self-efficacy. For example, where regimen self-efficacy was strongly associated with hospital setting, diet self-efficacy was more related to patient characteristics like age and education.

The present study did have some limitations. First, since our study recruited T2DM patients from only the central and northeastern region of Thailand, our sample may not be representative of all T2DM patients in similar Southeast Asia healthcare settings in general or even all areas of Thailand. The cross-sectional nature of our study design also leads to some limitations. First, for the criterion validity, our study design was restricted to assessing only concurrent validity. We demonstrated that several T-DMSES subscales were highly associated with HbA1c control measured at the same time. Ideally, DMSES should be assessed against HbA1c level collected at a future time to establish predictive validity. Furthermore, potential risk factors for poor DMSES were also measured concurrently with DMSES. Again, a cohort study would provide better evidence regarding the role patient characteristics play as risk factors in poor diabetes management self-efficacy. A third limitation stemming from the cross-sectional nature of our study design was that we were unable to assess the test-retest reliability of the T-DMSES; we could only collect one instance of patients' T-DMSES scores. Finally, for reasons of brevity, we restricted our analysis of patient characteristic's associations with T-DMSES subscales to bivariate analysis and leave the multivariable modeling of the T-DMSES for future study.

Our study also had some strengths. First, our validation of T-DMSES is one of the most comprehensive evaluations of the psychometric properties of the DMSES instrument ever conducted in any population. Our study was multicenter and captured the full spectrum of healthcare available in Thailand. All previous studies attempting to validate DMSES have used samples of between 88 to 440 patients, invariably collected from a single center. In our study, we collected 700 patients from 4 hospitals representing both rural and urban areas of two provinces.

## 5. Conclusion

Our study translates and appropriately validates the DMSES in the Thai T2DM patient population. T-DMSES was shown to have good psychometric properties including both construct and criterion validity. We also demonstrated T-DMSES to have strong internal consistency reliability, both overall and for most of its subscales. We also established that the T-DMSES is strongly indicative of HbA1c control in Thai T2DM patients. In the future, T-DMSES is likely to provide valuable insights into the epidemiology of diabetes management self-efficacy and may also be used to evaluate interventions to reduce poor self-care or improve the achievement of clinical targets, in T2DM patients, in turn, potentially reducing the incidence of, and mortality from, T2DM complications.

## Figures and Tables

**Table 1:** Patient characteristics.

Characteristics	*n* = 700
Hospital *n* (%)	
Phuphaman Hospital	60 (8.6)
Srinagarind Hospital	78 (11.1)
Wechkaroonrasm Hospital	242 (34.6)
Chulalongkorn Hospital	320 (45.7)
Gender *n* (%)	
Male	208 (29.7)
Female	492 (70.3)
Age (years) *n* (%)	
Mean (SD)	65.16 (10.9)
Range	26–95
Marital status *n* (%)	
Single	57 (8.1)
Married	465 (66.4)
Divorce	165 (23.6)
Separate	13 (1.9)

**Table 2:** Standardized factor loading of the T-DMSES.

Factors	Items	Diet	Monitor	Physical	Regimen
Diet (9 items)					
(i) DMSES4	I can choose to eat good and healthy foods that are beneficial to my health	0.607^┼^	—	—	—
(ii) DMSES5	I can choose to eat various foods to maintain a healthy diet plan	0.653	—	—	—
(iii) DMSES9	I can maintain a healthy diet plan in the event that I get sick	0.642	—	—	—
(iv) DMSES10	I can follow a healthy diet plan regularly	0.855	—	—	—
(v) DMSES13	I can follow a healthy diet plan even when I am not at home	0.897	—	—	—
(vi) DMSES14	I can choose from various foods to maintain a healthy diet plan when I am not at home	0.914	—	—	—
(vii) DMSES15	I can follow a healthy diet plan during festivals, traditions, or rituals	0.894	—	—	—
(viii) DMSES16	I can choose to eat various foods to maintain a healthy diet plan when I eat foods at parties	0.859	—	—	—
(ix) DMSES17	I can maintain a healthy diet plan when I am feeling stressed or worried	0.473	—	—	—
Monitor (4 items)					
(i) DMSES1	I can check blood glucose levels by myself if necessary	—	0.238^┼^	—	—
(ii) DMSES2	I can reduce blood glucose levels when glucose levels in my blood are too high (e.g., changing the kinds of foods I eat)	—	0.890	—	—
(iii) DMSES3	I can increase blood glucose levels when glucose levels in my blood are too low (e.g., changing the kinds of foods I eat)	—	0.309	—	—
(iv) DMSES7	I can attend to my feet (e.g., cutting toenails and taking care of myself not causing wounds)	—	0.302	—	—
Physical (4 items)					
(i) DMSES6	I can control my body weight and maintain appropriate weight ranges	—	—	0.405^┼^	—
(ii) DMSES8	I can exercise and perform sufficient physical activity (e.g., walking, aerobic dancing, and muscle exercise)	—	—	0.560	—
(iii) DMSES11	I can increase the amount that I exercise if a doctor advises me to do so	—	—	0.766	—
(iv) DMSES12	In the case that I exercise more, I can modify my healthy diet plan	—	—	0.890	—
Regimen (3 items)					
(i) DMSES18	I can schedule an appointment to see a doctor four times a year to check my diabetes	—	—	—	0.677^┼^
(ii) DMSES19	I can take medicines as prescribed by a doctor	—	—	—	0.841
(iii) DMSES20	I can keep taking medicines continuously when I am sick	—	—	—	0.757

^┼^Item constraint (no significant test conducted).

**Table 3:** Interfactor correlation of T-DMSES

	Monitor	Physical	Regimen
Diet	0.767	0.538	0.204
Monitor	—	0.509	0.265
Physical	—	—	0.170

All interfactor correlations were statistically significant (*p* < 0.001).

**Table 4:** Sensitivity, specificity, positive and negative predictive values (PPV and NPV), and the positive and negative likelihood ratios (LR+ and LR−) of the T-DMSES subscale association with HbA1c control.

	Sensitivity	Specificity	PPV	NPV	LR+	LR−
DMSES diet	0.86	0.79	0.79	0.86	4.13	0.18
DMSES monitor	0.83	0.76	0.76	0.83	3.44	0.23
DMSES physical	0.55	0.77	0.68	0.65	2.39	0.58
DMSES regimen	0.71	0.80	0.76	0.75	3.47	0.37
DMSES total	0.81	0.84	0.82	0.83	5.02	0.23

**Table 5:** Cronbach's alpha of each subscale of T-DMSES.

Scales	Cronbach's alpha	95% CI
Diet	0.92	0.90, 0.94
Monitor	0.45	0.36, 0.53
Physical	0.71	0.65, 0.77
Regimen	0.80	0.73, 0.87

**Table 6:** Crude odds ratios and (95% confidence intervals) from ordinal logistic regression analysis of patient characteristic for each DMSES subscales.

Effect	OR_Diet_	OR_Monitor_	OR_Physical_	OR_Regimen_
Province (ref: Bangkok)				
Khon Kaen	0.67 (0.47–0.96)	0.99 (0.68–1.43)	1.36 (0.91–2.03)	0.31 (0.18–0.52)
Hospital size (ref: big hospital)				
Small hospital	0.95 (0.71–1.27)	0.97 (0.72–1.31)	0.92 (0.68–1.29)	0.47 (0.28–0.79)
Sex (ref: male)				
Female	1.16 (1.17–2.22)	0.95 (0.68–1.32)	1.01 (0.71–1.43)	1.73 (1.03–2.91)
Marital status (ref: single)	*χ*_LRT_^2^ = 6.48^∗^	*χ*_LRT_^2^ = 0.16	*χ*_LRT_^2^ = 2.92	*χ*_LRT_^2^ = 2.27
Married	0.71 (0.41–1.23)	1.11 (0.64–1.93)	0.61 (0.34–1.09)	1.07 (0.45–2.58)
WDS	1.08 (0.60–1.95)	1.09 (0.60–1.96)	0.59 (0.31–1.11)	1.70 (0.62–4.61)
Education (ref: no formal education)	*χ*_LRT_^2^ = 15.37^∗∗^	*χ*_LRT_^2^ = 2.18	*χ*_LRT_^2^ = 5.07	*χ*_LRT_^2^ = 7.77
Primary	0.36 (0.20–0.65)	0.84 (0.46–1.54)	1.32 (0.70–2.50)	0.39 (0.09–1.69)
Secondary	0.29 (0.15–0.55)	1.04 (0.54–2.00)	1.27 (0.63–2.55)	0.20 (0.04–0.92)
Bach+	0.30 (0.15–0.59)	1.09 (0.56–2.14)	2.00 (0.98–4.08)	0.27 (0.06–1.28)
Religion (ref: Buddhism)				
Non-Buddhism	1.02 (0.73–1.42)	1.04 (0.73–1.47)	0.86 (0.59–1.26)	0.64 (0.37–1.13)
Income (ref: <5K)	*χ*_LRT_^2^ = 6.31	*χ*_LRT_^2^ = 2.59	*χ*_LRT_^2^ = 4.54	*χ*_LRT_^2^ = 10.66^∗∗^
5–9.99K	0.85 (0.55–1.32)	1.12 (0.71–1.77)	1.23 (0.75–2.00)	0.44 (0.20–0.96)
10–14.99K	0.75 (0.48–1.18)	1.19 (0.74–1.92)	1.43 (0.85–2.38)	0.43 (0.19–0.97)
15–24.99K	0.91 (0.58–1.43)	1.37 (0.87–2.17)	1.58 (0.96–2.59)	0.50 (0.22–1.09)
25+K	0.59 (0.38–0.90)	1.28 (0.82–2.01)	1.27 (0.78–2.06)	0.35 (0.17–0.72)
BMI (ref: healthy)	*χ*_LRT_^2^ = 9.04^∗^	*χ*_LRT_^2^ = 10.16^∗^	*χ*_LRT_^2^ = 3.54	*χ*_LRT_^2^ = 0.03
<18.5	1.30 (0.55–3.09)	2.56 (1.06–6.18)	0.63 (0.23–1.70)	0.89 (0.20–3.86)
25–29.9	0.63 (0.45–0.90)	0.77 (0.53–1.10)	0.82 (0.56–1.19)	1.01 (0.55–1.84)
30+	0.68 (0.47–0.98)	0.70 (0.48–1.02)	0.69 90.46–1.04)	1.01 (0.53–1.89)
Family history of DM (ref: no)				
Yes	0.74 (0.55–0.99)	0.86 (0.64–1.16)	1.09 (0.79–1.50)	0.78 (0.47–1.29)
DM treatment (ref: diet and exercise)	*χ*_LRT_^2^ = 69.10	*χ*_LRT_^2^ = 46.97	*χ*_LRT_^2^ = 33.14	*χ*_LRT_^2^ = 2.10
OHA	0.39 (0.13–1.17)	0.24 (0.08–0.74)	0.62 (0.19–1.96)	0.21 (0.01–3.98)
Ins	0.11 (0.03–0.37)	0.10 (0.03–0.33)	0.24 (0.07–0.82)	0.16 (0.01–3.23)
OHA + Ins	0.11 (0.03–0.34)	0.08 (0.02–0.27)	0.23 (0.07–0.76)	0.18 (0.01–3.46)
Smoking (ref: no)	*χ*_LRT_^2^ = 5.07	*χ*_LRT_^2^ = 4.14	*χ*_LRT_^2^ = 0.06	*χ*_LRT_^2^ = 5.90
Previous	0.64 (0.41–0.99)	0.92 (0.59–1.45)	0.95 (0.58–1.57)	0.88 (0.41–1.89)
Current	1.41 (0.65–3.02)	2.26 (1.01–5.04)	0.91 (0.36–2.25)	0.26 (0.10–0.69)
Alcohol (ref: no)	*χ*_LRT_^2^ = 7.01^∗^	*χ*_LRT_^2^ = 1.52	*χ*_LRT_^2^ = 0.11	*χ*_LRT_^2^ = 6.85^∗^
Previous	0.59 (0.38–0.91)	0.96 (0.62–1.51)	0.95 (0.58–1.54)	0.44 (0.23–0.84)
Current	0.63 (0.34–1.18)	1.49 (0.77–2.87)	0.90 (0.46–1.79)	0.50 (0.20–1.23)
Comorbid (ref: no)				
Yes	1.07 (0.58–1.96)	0.94 (0.50–1.74)	0.81 (0.41–1.58)	2.09 (0.90–4.89)
Age (ref: ≤10)				
>10 years	1.31 (1.15–1.50)	1.06 (0.92–1.21)	0.90 (0.78–1.04)	1.85 (1.46–2.34)
Duration of DM (ref: <5 years)				
≥5 years	1.25 (0.98–1.52)	1.02 (0.89–1.23)	0.87 (0.68–1.12)	1.47 (1.18–1.86)

^∗^ *p* < 0.05; ^∗∗^*p* < 0.01.
